# Analysis of geographic location and pathways for influenza A virus infection of commercial upland game bird and conventional poultry farms in the United States of America

**DOI:** 10.1186/s12917-019-1876-y

**Published:** 2019-05-14

**Authors:** Amos Ssematimba, Kaitlyn M. St. Charles, Peter J. Bonney, Sasidhar Malladi, Marie Culhane, Timothy J. Goldsmith, David A. Halvorson, Carol J. Cardona

**Affiliations:** 1 Secure Food Systems Team, College of Veterinary Medicine, University of Minnesota, 1971 Commonwealth Avenue, Saint Paul, MN 55108 USA; 2 Department of Mathematics, Faculty of Science, Gulu University, P.O. Box 166, Gulu, Uganda

**Keywords:** Influenza a virus, Infection pathways, Epidemiological contacts, Biosecurity, Upland game birds

## Abstract

**Background:**

Avian influenza (AI) is an infectious viral disease that affects several species and has zoonotic potential. Due to its associated health and economic repercussions, minimizing AI outbreaks is important. However, most control measures are generic and mostly target pathways important for the conventional poultry farms producing chickens, turkeys, and eggs and may not target other pathways that may be specific to the upland game bird sector. The goal of this study is to provide evidence to support the development of novel strategies for sector-specific AI control by comparing and contrasting practices and potential pathways for spread in upland game bird farms with those for conventional poultry farms in the United States. Farm practices and processes, seasonality of activities, geographic location and inter-farm distance were analyzed across the sectors. All the identified differences were framed and discussed in the context of their associated pathways for virus introduction into the farm and subsequent between-farm spread.

**Results:**

Differences stemming from production systems and seasonality, inter-farm distance and farm densities were evident and these could influence both fomite-mediated and local-area spread risks. Upland game bird farms operate under a single, independent owner rather than being contracted with or owned by a company with other farms as is the case with conventional poultry. The seasonal marketing of upland game birds, largely driven by hunting seasons, implies that movements are seasonal and customer-vendor dynamics vary between industry groups. Farm location analysis revealed that, on average, an upland game bird premises was 15.42 km away from the nearest neighboring premises with birds compared to 3.74 km for turkey premises. Compared to turkey premises, the average poultry farm density in a radius of 10 km of an upland game bird premises was less than a half, and turkey premises were 3.8 times (43.5% compared with 11.5%) more likely to fall within a control area during the 2015 Minnesota outbreak.

**Conclusions:**

We conclude that the existing differences in the seasonality of production, isolated geographic location and epidemiological seclusion of farms influence AI spread dynamics and therefore disease control measures should be informed by these and other factors to achieve success.

**Electronic supplementary material:**

The online version of this article (10.1186/s12917-019-1876-y) contains supplementary material, which is available to authorized users.

## Background

Although avian influenza (AI) is known to wreak havoc on the national economy when large outbreaks occur, its impact on individual poultry farmers is enormous due to the threat to their wellbeing, social security and economy. Identifying/managing mechanisms of influenza A virus (IAV) introduction onto and spread between farms is essential to control the disease. It is hypothesized that upon introduction from wild aquatic avian reservoir hosts [1–5], complex farm operational networks facilitate the transmission of virus via direct or indirect contact with infectious material [6–9].

While some exposure pathways are well-known and are common between poultry sectors, we hypothesize that complex species- or sector-specific pathways exist and may contribute differently to between-farm AI spread. In an analysis of historical AI epizootics in the United States (U.S.) between 1980 and 2017, St. Charles et al. [10] reported that 23 epizootics involved commercial raised-for-release upland game birds (hereafter called upland game birds), only 14% of which involved multiple premises. On the other hand, twenty-fold more (485) epizootics were documented in commercial poultry farms raising turkeys and chickens with 42% involving multiple premises. Such data illustrate that it is possible for an individual sector to be more or less prone to having IAV introductions and/or spread. During the 2015 highly pathogenic AI (HPAI) outbreak in Minnesota, 94.5% (104/110) of cases involved turkey premises [11]. Undoubtedly, that is at least partially because Minnesota is the top producer of turkeys in the nation. However, no upland game bird farms were infected despite Minnesota also being among the top pheasant producing states. It is important to note that turkeys and pheasants are closely related [12] with similar susceptibilities to IAV.

Sector-specific transmission patterns in both recent and historical epizootics are potentially indicative of inherent differences in exposure pathways. We aim to illustrate that AI exposure pathways are likely different between poultry species and sectors in the U.S. Based on the evidence pointing to limited inter- and intra-sector AI spread [10], the upland game bird farm sector was selected to explore the hypothesis of sector-specific AI spread networks.

Although the upland game bird industry is economically and epidemiologically relevant to AI ecology, only limited information is available for this sector. This poses more challenges to understanding the mechanisms of disease spread and consequently its efficacious control. Moreover, the U.S. Department of Agriculture (USDA), in its poultry industry manual [12], recognizes that the unique aspects in game bird production merit consideration during outbreaks of contagious foreign animal diseases like HPAI.

Here, we compare the factors known to impact disease spread across this sector and the combined conventional meat and egg poultry industries (hereafter called conventional poultry). Specifically, farm practices and processes, seasonality of activities, and geographic locations, and inter-premises distances are analyzed across these sectors. We highlight practices that are different (using absent vs present comparison structure) between conventional poultry and upland game bird industry groups and frame the day-to-day differences in production systems in the context of day-to-day contacts that could result in viral transmission. In addition, we qualitatively analyze how the specific aspect of seasonal production relates to outbreaks in the targeted sectors. Lastly, we analyze premises locations to infer their fates during the 2015 HPAI epizootic in Minnesota, U.S. as a case study.

## Results

### Production practices assessment

Several key differences that separate the upland game bird industry from conventional poultry industries were identified and details of the general outcomes of the comparison are presented in Additional file 1 :Table S1. Below we highlight some of the key findings for each production practice category.

#### Husbandry-related aspects

**Fig. 1:**
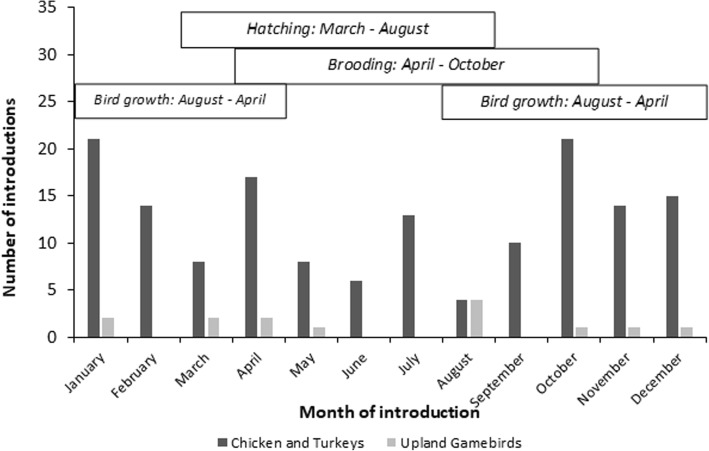
Distribution of historical monthly IAV introductions in United States poultry from 1980 to 2017 in the commercial raised-for-release upland game bird industry and combined conventional poultry industries including meat (chicken and turkey) and egg laying (laying chickens, pullets, breeder chickens, breeder turkeys) birds. Seasonal production activities of the commercial raised-for-release upland game bird industry are shown in boxes above the graph

Twenty-four of the 34 practices (see Additional file 1 :Table S1) studied were determined to be different between industry groups and 10 were similar. Commercial upland game bird farms operate under a single, independent owner rather than being contracted with or owned by a company with other farms. Upland game bird farms operate as complete single-site production premises (i.e., birds are bred, hatched, brooded, and grown to maturity by a single establishment often on a single premises). In contrast, conventional poultry farms mostly specialize in one stage of production [12, 13]. Compared to market age conventional poultry, the vast majority of upland game birds are outdoors in pens in smaller groups and lower density than typical of conventional poultry. For example, compared to breeder turkeys whose maximum area per bird is for > 15 weeks old breeder toms at 8 to10 ft^2^/bird, breeder pheasants are raised at 25 to 30 ft^2^/bird [12]. On upland game bird farms, different production stages (e.g., breeding, hatching, brooding, growing) occur seasonally as illustrated in Fig. 1. Consequently, pen downtime for these farms was reported to range from two to eight months in comparison with the typical two weeks or less in conventional poultry industries [12].

#### Market-related aspects

Thirteen of the 14 marketing-related practices were determined to be different between the industry groups. The seasonal marketing of upland game birds (i.e., seasonal customer-base), largely driven by hunting seasons, greatly impacts movements of birds for sale, with movements only occurring during certain months of the year. Additionally, customer-vendor dynamics vary between industry groups. It is observed that in the upland game bird industry, one supplier (i.e., one farm) may exclusively supply multiple terminal customers (i.e., hunting preserves) while for conventional poultry, multiple suppliers (i.e., multiple contracted or company owned farms) supply one terminal customer (e.g., one processing plant). In regards to customer bird-requirements, all upland game bird farms engage in partial-farm and -flock removals due to the smaller batches of birds demanded by customers. In contrast, for conventional poultry, the entire flock is marketed at once in an all-in-all-out system. Additionally, upland game birds have to travel long distances (100 to 1000 miles) to customers because of the isolated locations of hunting preserves and the upland game bird farms.

#### Fomite-related aspects

Eight of nine personnel-related practices were different between the industry groups. It was reported that, in most regions of the U.S., contracted veterinarians working with upland game bird farms were neither likely to work with other upland game bird nor other poultry premises in contrast to the common practice of sharing labor on conventional poultry farms [13]. For day-to-day farm activities, while it is common practice to hire extra labor for special jobs (hereafter called crews) on conventional poultry farms, individual employees performed almost all farm tasks (e.g., bird-catching, cleaning and disinfection, land maintenance, etc.) on upland game bird farms. Equipment and vehicle practices may serve as fomites for pathogen spread. Five out of the seven equipment-related practices were different between industry groups. Of the vehicle-related practices, only one of the three practices was different between industry groups. Upland game bird farms reportedly own all equipment used on farm and the vehicles used to move birds. On the other hand, equipment and vehicle sharing is a common practice in the other industries. See Additional file 1 :Table S1 for more details and exact differences.

### Temporal assessment of IAV introductions

A summary of historical IAV introductions by month in the U.S. from 1980 to 2017 is presented in Fig. 1. Only 36% of the introductions in upland game birds occurred between September and January (i.e., U.S. fall and winter seasons) compared to 63% in other poultry industries for the same period. No introductions in upland game birds with documented months of introduction were reported in February, June, July and September to date. The majority of reported outbreaks (93%) in commercial upland game birds occurred during the season of mature market-bird production (August through April). Additionally, 29% of outbreaks occurred during August, the first month of the growing season and the month in which there is overlap between chick hatching season, brooding season, and mature bird production season.

### Identified factors influencing AI dynamics on commercial upland game bird farms

#### Protective factors

Some observed differences in production practices in the upland game bird industry were attributed to physical- or location-based isolation, specifically relating to the fact that upland game bird farms are geographically isolated. Upland game bird producers reported that other poultry or upland game bird farms are too far away to render resource (equipment, vehicle, and personnel) sharing as a convenient or useful practice (Secure Upland Gamebird Supply Plan Working Group, pers. comm.). Consequently, the geographic isolation decreases the likelihood of spreading virus on fomites.

Not only are upland game bird farms geographically isolated, they are also operationally and socially isolated. Typical commercial upland game bird producers almost always own a single premises with complete production (e.g., breed-hatch-grow) occurring all on one site [12]. This eliminates producer participation in the operational networks associated with larger vertically integrated companies typical of other poultry industries and thus limits avenues for sharing resources.

Seasonal isolation was also evident for upland game bird farms. The extended downtime between production cycles increases the possibility that the farms have susceptible birds when introductions occur in conventional poultry. In other words, the observed sector-specific production seasonality narrows the length of the exposure window as well as the contact network that is instrumental in disease spread.

The upland game bird industry’s market structure eliminates additional points of spread because the end destination of birds is usually only receiving birds from one producer, creating market-based isolation. Such isolation eliminates chances of cross contamination between farms that can occur at end destinations for the conventional poultry commodities (e.g., processing plants).

#### Increased risk factors

Despite having location-based epidemiological isolation, location-based transmission risks were identified. The remote locations of upland game bird farms and also their hunting preserve customers results into transporting birds over long distances (up to 1000 miles) and this could increase potential spread of disease via local area spread and during transit. Operationally, the common upland game bird industry-practice of raising birds outside exposes the birds to an open-air environment that is directly shared with wild birds and other mammals. As is illustrated in Additional file 2 :Table S2, there are 14 production practices that only occur with the use of outdoor pens. This system may create challenges related to cleaning and disinfection, air quality control, wild bird and predator control, and line of separation protocol feasibility.

Season of production also poses a risk as it is possible that the overlap of the start of the upland game bird production season and fall wild bird migration patterns increase the likelihood of virus introduction [14]. This is supported by the fact that 4 of the 14 upland game bird historical IAV introductions (29%) occurred in August (Fig. 1). Note that this peak correlates directly with the reported peaking of avian influenza virus prevalence in free-living birds [15, 16].

### Minnesota 2015 spatial analysis

Location data was obtained for 774 poultry premises in Minnesota, including 26 game bird and 398 turkey premises. A summary of the results from the survivability analysis of control areas, density and inter-farm distance assessment during the 2015 Minnesota HPAI epizootic is presented in Table 1. It is observed that, on average, a commercial upland game bird premises was 15.42 km away from the nearest neighboring premises with birds compared to 3.74 km for commercial turkey premises. The average poultry farm density in a radius of 10 km of a commercial upland game bird premises was less than a half of that of commercial turkey premises, and turkey premises were 3.8 times (43.5% compared with 11.5%) more likely to fall within a control area. Of those that fell within a control area, none (i.e., 0/3) of the upland game bird and 36.4% (i.e., 63/173) of the turkey premises ultimately were infected.

**Table 1:** The average minimum distance between premises, the average number of premises within in 10 km of each premises as well as the number of premises in a control area and their ultimate disease status for game bird and turkey premises

	Average minimum distance (km)		Average number of premises within 10 km of each premises	Number of premises in a control area		
	To any premises with birds	To same sector premises		Infected	Never infected	All (%)
Game birds (Total = 26, Number infected = 0)	15.42	32.47	4.6	0	3	3/26 (11.5%)
Turkeys (Total = 398, Number infected = 98^a^)	3.74	4.15	9.7	63	110	173/398 (43.5%)

^a^Among the infected turkey premises, location data was available for 98

## Discussion

While there is the perception of higher risk of IAV introduction on upland game bird farms because of their outdoor production [17], the risk of secondary spread from these facilities appears to be much lower [10]. The findings of this study suggest that production seasonality and geographical- and epidemiological-seclusion of upland game bird premises may contribute to reduced AI spread patterns compared to turkey and chicken production systems. Since the potential pathways of virus spread are largely governed by variable farm practices and activities [7, 18–22], mitigation measures and policy around outbreak management need to be informed by sector-specific details leading to these pathways.

The observed differences between the compared industry sectors stemming from production/husbandry systems, seasonality of production, inter-premises distance and farm densities could influence both fomite-mediated and local area spread risks. These factors have been implicated in previous outbreaks and general analyses, for example, during the 2014–2015 U.S. HPAI outbreak in which sharing of equipment and farm workers and visitors in poultry sectors were identified as likely contributors to virus spread [11, 23].

In our analysis of patterns of introductions, it was evident that the majority of introductions in the conventional industry occur in fall-winter while those in the upland game bird sector occur in spring-summer. Such observations may be connected to the seasonal aspect of having large numbers of birds outdoors in late summer in preparation for hunting seasons. Additionally, the findings that, of all IAV introductions on upland game bird farms, 29% occurred in August when there is an overlap between bird growth, brooding and hatching and 93% (13/14) occurred during mature bird production are of epidemiological interest. Given that these introductions occur across the continental U.S., it may be of use to explore the correlation between introductions, the timeline of the upland game bird production system, and regional weather variation. Because weather phenomena can impact virus survival among others, it is possible that the additional factor of weather could cause variation among introduction occurrences alongside production periods where more or less birds are present on the farm and thus warrants investigation.

Historically, in Minnesota at least, peak avian influenza virus prevalence in wild birds occurs in late July to August [14–16]. Thus, investigations into the emptying of pens in relation to, for example, wild bird migration [5] could provide more insight. Note that during the 2015 U.S. epizootic, most outbreaks occurred in April [5]. On seasonality at the national level, there are regional differences in wild bird migration and consequently hunting seasons since the two often overlap. This regional synchronization of migration and hunting means that the identified wild bird migration-associated risk factors are likely to be similar across the nation.

In general, the mechanisms of seasonality in disease dynamics may also include cycles in pathogen appearance or virulence, cyclic occurrence of activity, human/host behavior and climatic cycles [24, 25] as well as pathogen survivability. Additionally, seasonal variations in mortality rates and pest control methods [26] as well as wild bird migration patterns [1–4, 27], and market cycles should be considered.

Our analysis of the 2015 situation in Minnesota revealed that upland game bird farms were usually located in areas with lower poultry farm density, located much farther away from any other premises with birds and were consequently less likely to be in a control area. This geographical isolation indicates that these farms generally have a lower risk of local area spread in disease outbreaks. The potential role of farm density on AI dynamics is highlighted by Bonney et al. [28] who, upon assessing distance-dependent risk of AI transmission between farms, found that early marketing (which lowers the density of susceptible farms) may have played a role in controlling the 2015 outbreak in Minnesota.

Interestingly, when compared to conventional poultry, the results from our concurrent analyses may illustrate that while the upland game bird sector may lack some areas of structural biosecurity such as having secure housing with appropriate bird and rodent proofing measures [29], the sector is stronger in other structural biosecurity aspects e.g., management approaches [30] such as vertically integrating production on a premises rather than at an industry level. Additionally, our combined results illustrate inherently advantageous conceptual biosecurity [29] for upland game bird farms such as the greater geographical isolation of farms depicted in Table 1. This isolation may not only contribute to reduced local area spread risks, but also enhances social and epidemiological isolation due to lack of nearby farms with which to share resources or services. Improved understanding of individual sector strengths in biosecurity frameworks (i.e., whether conceptual, structural, or operational) is needed for a better understanding of exposure pathways within sectors and consequently to guide the development of more effective sector-specific disease control strategies.

Note that our premises‘location analysis focused on the state of Minnesota due to its heavy involvement in the 2014–2015 U.S. HPAI outbreak and the accessibility and format of its outbreak data. Moreover, Minnesota is among the top producers of both pheasants and turkeys [12]. Other limitations encountered included those during historical introduction analysis specifically because not all historical epizootics had a registered month of introduction which led to use of a subset of epizootics (31% of historical epizootics in conventional poultry and 61% of historical epizootics in upland game birds) analyzed.

While performing our sector-specific pathway analysis, we encountered few up-to-date references depicting the modern commercial raised-for-release upland game bird industry. Furthermore, limited available data did not distinguish between hobby, small producers, and commercial raised-for-release producers. Thus, we partly relied on semi-structured group interviews with the SUGS. When needed, we also utilized expert opinion. Both the paucity of historical farm characteristic data and our approach of obtaining information from interviews and expert opinions may have limitations. On the within-sector farm-specific differences in practices, only a few existed and these were mainly on matters of “how” and not “whether” a practice existed. Moreover, the difference in how a given activity was performed does not influence the outcomes of our “present vs absent” approach in relation to HPAI spread.

This study aimed at comparing production practices in conventional vs upland game bird sectors with the ultimate aim of inferring how these practices may influence the transmission dynamics of avian influenza virus between premises. Practices were analyzed on the basis of “present vs absent” and no detailed statistical analysis is permissible for such an approach. A study that compares frequencies of different practices would generate more quantitative information for which detailed statistical analyses would be permissible. However, the extrapolation from the frequency of a practice to the risk of avian influenza transmission is never straight forward since the relationship is likely nonlinear. Our approach answers the research questions of the current study concisely since presence of a relevant practice would imply non-zero probability of virus transmission via that practice while absence would imply zero probability. This straight forward translation into risk was deemed advantageous.

## Conclusion

The commercial upland game bird sector is an economically significant sector in the poultry industry and is of epidemiological relevance during disease outbreaks. Despite all this, most studies, for example [13, 31–34], have largely focused on conventional poultry leaving a knowledge gap of the upland game bird sector and IAV transmission dynamics. Our findings begin to address this knowledge gap, and reveal that, while upland game bird farms, compared to conventional poultry, may have extra environmental IAV exposure risks, some of their practices exclude them from other key exposure pathways. Additionally, there is limited overlap in practices between the upland game bird industry and the conventional turkey and chicken industries. While the overlapping practices may be few, analyzing those specific practices will help determine where and when spread could happen between sectors. Consequently, adjusting existing control measures to cater for sector-specific differences will improve management of future AI epizootics and perhaps those involving other pathogens.

There is potential for improvement at all the three levels of biosecurity namely, conceptual, structural and procedural biosecurity. Under conceptual biosecurity, upland game bird farms fared better at one component namely, physical isolation of the premises from other poultry premises while conventional poultry farms were better suited to control HPAI introduction by vermin and wild animals. Much as close proximity to other poultry operations may have its own logistical advantages, in the context of preventing the economically devastating HPAI introduction onto the premises, prospective conventional poultry farmers should choose locations in areas with less density of poultry. Additionally, producers should always consider a tradeoff between reducing operation costs e.g., by sharing equipment and the increased risk of exposure to HPAI.

Although the absent vs present-based comparison methodology is enough to achieve this study’s objectives, it should serve only as a foundation and future work should delve into a deeper level of comparison e.g., frequency of occurrence, and also compare individual sectors (i.e., broilers, turkeys, layers, and upland game birds) to eliminate potential generalization of practices in cases where commodity (and hence operational) differences exist. Note that, although free-range or pasture-raised poultry were not a part of this study, some of their husbandry practices might in some respects resemble those in commercial upland game bird production more than conventional production. Such comparisons between free range or pastured range poultry systems and upland game bird production systems should be investigated in future studies.

## Methods

### Production and management systems

#### Data collection

Information on relevant practices (i.e., those reported in literature [13, 31, 33, 35], to impact AI spread dynamics) for conventional industries was obtained from the USDA authored Poultry Industry Manual [12] and from the pool of literature used within Secure Poultry Systems Risks Assessment Background sections [36]. Information pertaining to commercial upland game birds was pulled from the Poultry Industry Manual as well as industry-focused articles. Industry articles were selected by searching the published materials listed by the cooperative extension agencies of universities of top pheasant producing states [37] including Kansas, Minnesota, Ohio, Pennsylvania, South Dakota, and Wisconsin. The search strategy yielded 74 articles, however, only articles describing production of commercial upland game birds for sale with accessible publication dates published within the last 20 years were used to produce the most current picture of the industry. Thus 69 articles were excluded. For variables related to emergency practices, case studies [38] and USDA policy was referenced [39]. Additional information was gathered using semi-structured group interviews to active producers from the Secure Upland Gamebird Supply Plan (SUGS) working group (consisting of North American Gamebird Association representatives, commercial upland game bird farm owners and managers, and regulatory veterinarians) during work group meetings. The SUGS working group provided missing information and assisted in the clarification on some otherwise unclear aspects of industry practices.

#### Data analysis

The production practices assessed were categorized as husbandry/management practices (inputs and operational processes), market practices (outputs), or personnel deployment, equipment usage, and vehicle usage (fomites). Those compared are listed in Additional file 2 :Table S2. Production practices for targeted industry sectors were determined to be either absent or present based on published materials or subject matter expert interviews, as described. Based on absent or present methods to fulfill production practice categories, different types of pathways between the upland game bird industry and the combined conventional poultry were identified. Note that this discrete categorization of data was done because assessment of the degree of risk associated with pathways that both industry groups possess was not the aim. The resulting data were qualitatively assessed in the context of how day-to-day activities in the target industry sectors influence the transmission dynamics of IAV based upon current understanding of pathogen pathway analysis. The results were descriptively framed via potential exposure pathways that would likely increase or decrease the risk of IAV infection based on current understandings of AI spread dynamics in the commercial upland game bird industry.

### Seasonality of production activities and IAV introductions

We compared the seasonality of production activities across the industry sectors in the entire U.S. bearing in mind the possibility of region variation. Thus, it is recognized that different geographical regions will have variability in hunting seasons and subsequent mild variability in production stages (e.g., southern states starting and ending later into fall and winter). Within in this context seasonality is defined as the time of year rather than other factors associated with season such as weather.

We further aligned seasonal production practices with historical IAV introductions for the upland game bird sector. For this purpose, a list of documented historical IAV introductions reported by St. Charles et al. [10] was utilized among other data. Month of detection for each outbreak was derived from published literature. Only data that included the month of detection were used in the analysis. A reported case was counted as an epizootic if it was caused by an IAV with a unique subtype or it was isolated from other cases in time and space. If cases caused by the same viral subtype were reported from the same state and detections were reported at least two months apart, they were considered separate introductions for the purposes of this study. For all the outbreaks with complete information, the monthly distribution of epizootics was summarized and compared across industry sectors and only descriptive statistics were utilized due to data limitations and nature of the research questions.

### Farm locations during the Minnesota 2015 HPAI epizootic

#### Scope

The geographical distribution of poultry premises (inferred from premises’ location data) and control areas (i.e., areas surrounding infected premises where disease surveillance and other disease control measures are intensified to ensure early disease detection and containment of the outbreak) were collected for turkey and upland game bird premises in Minnesota in 2015 for inter-premises distance analysis. Minnesota was selected based on the availability of complete data in the desired format and the fact that it was heavily involved in the 2014–2015 United States HPAI outbreak. This analysis focused on determining how many premises of each type were located within control areas, how many became infected or not during the 2015 outbreak and how the average minimum inter-premises distances varied between premises by type.

#### Data and its analysis

Premises location data (i.e., the latitude and longitude coordinates) were obtained for all commercial poultry premises in Minnesota in 2015. In the control area analysis, we were interested in determining the number of premises that fell within a control area at any time of the 2015 outbreak. Any premises was considered located within a control area if it was 10 km or closer from at least one infected premises. The 10 km radius, measured using Euclidean distance, was chosen in order to conform to the current guidelines for HPAI control in the United States. For an ultimately infected premises to be in a control area, we also required that its date of detection was strictly after that of the reference infected premises.

To determine the area spanned by a given infected farm’s control area and ultimately establish the number of farms therein, a 10 km circle was drawn around each HPAI virus positive premises on the day of detection until 21 days after completion of depopulation/disposal of all poultry on the infected premises. Twenty one days were selected in accordance with the HPAI control guidelines for control area degazettement. The number of premises of each type (i.e., turkeys and upland game birds) in a control area were determined and grouped based on their ultimate infection status.

Non-outbreak spatial distribution of farms in Minnesota was used to descriptively determine the mean number of poultry premises within 10 km of a turkey or an upland game bird premises, the average minimum distance between premises of the same type, and the average minimum distance between premises of any type. Specifically, the average minimum inter-premises distance was estimated by finding the closest premises (using Euclidean distance measure) among all premises of the target type for each of the game bird or turkey premises and then averaging those distances.

## Additional files

Additional file 1: **Table S1.** Complete comparison* of industry group (i.e., commercial upland game bird industry and conventional poultry) practices derived from literature and subject matter experts. *Note that while frequencies and detailed observations were recorded, the qualitative analysis solely focused upon absence vs presence of different practices rather than the frequencies related to specific practices. The data within this supplementary table are the qualitative results for each industry group for the categories and subcategories listed in Additional file 2: :**Table S2.** Data was determined via semi-structured interviews with the Secure Upland Gamebird Supply Plan Working Group as well as literature (more details provided in methods). Data is provided in the form of qualitative descriptive statements describing the practices. (DOCX 15 kb)

Additional file 2: **Table S2.** List of production practices compared between industry groups (i.e., commercial upland game bird industry and conventional poultry). The data within this supplementary table outlines which production practices that were compared between industry groups, broken down into the categories of Husbandry, Marketing, Personnel, Equipment, and Vehicles. Within each category, subcategories are defined as appropriate. Categories and subsequent subcategories were derived from information outlined in the Poultry Industry Manual [12]. (DOCX 36 kb)

## Abbreviations

AI: Avian Influenza
HPAI: Highly Pathogenic Avian Influenza
IAV: Influenza type A Virus
U.S.: United States
USDA: United States Department of Agriculture
