# Supplementary material for: Analysis of geographic location and pathways for influenza A virus infection of commercial upland game bird and conventional poultry farms in the United States of America
Source: BMC Vet Res. 2019 May 14;15:147. doi: 10.1186/s12917-019-1876-y (PMC6518635; doi:10.1186/s12917-019-1876-y)
Supplement: Supplementary file 1 — Table S1. Complete comparison* of industry group (i.e., commercial upland game bird industry and conventional poultry) practices derived from literature and subject matter experts. *Note that while frequencies and detailed observations were recorded, the qualitative analysis solely focused upon absence vs presence of different practices rather than the frequencies related to specific practices. The data within this supplementary table are the qualitative results for each industry group for the categories and subcategories listed in Additional file 2: :Table S2. Data was determined via semi-structured interviews with the Secure Upland Gamebird Supply Plan Working Group as well as literature (more details provided in methods). Data is provided in the form of qualitative descriptive statements describing the practices. (DOCX 15 kb) [file 12917_2019_1876_MOESM1_ESM.docx]

Table S1. List of production practices compared between industry groups (i.e. commercial upland game bird industry and conventional poultry)

| **Husbandry** | |
| --- | --- |
| Breeding Components | |
| - Breeder Housing | - Breeder Location |
| Hatching Egg Components | |
| - Collection of Hatching Eggs | - Examination of Eggs |
| - Care/cleaning of Hatching Eggs | - Transfer of Eggs to Hatchery |
| - Incubation process | - Hatchery set up |
| Brooding Components | |
| - Sources of breeders | - Brooding set up (heat sources, light/ventilation, brooder prep) |
| - Brooder building location | - Use of litter in brooding |
| - Age groups in brooder premises |  |
| Grower Housing Components | |
| - Growing Housing set up | - Artificial lighting usage |
| - Use of Cover Crops | - Density |
| - Use of litter in growing birds | - Air Quality Control/Ventilation Control |
| - Water Fountains and Feeders | - Temperature Control |
| Feed | |
| - Type of feed | - Feed refilling method |
| - Feed source |  |
| Operations | |
| - Movement of birds from brooder set up to grower set up | - Bird Depopulation Methods |
| - Bird Catching Method | - Dead Bird Disposal |
| - C&D Process between batches of birds | - Integration of Production Stages in industry |
| - Complete C&D process used for post depopulation | - **Seasonal production** |
| Pest Control | |
| - Rodent Control | - Predator Control |
| - Wild bird Control |  |
| **Marketing** | |
| - Turnover rate of market bird flocks | - Market channel for live-birds (End destination for sold birds) |
| - Turnover rate in brooders | - Number of customers/contracts |
| - Number of premises involved in production system chain (egg to adult bird) | - Seasonal customer base |
| - Adding additional birds to pens or houses | - Number of other vendors dropping off at bird delivery site |
| - Partial farm removals | - Market channel for live-birds (End destination for sold birds) |
| - Partial flock removals | - Number of customers/contracts |
| - Flock/house/pen down time | - Seasonal customer base |
| - Distances birds are transported to market | - Onsite purchases of birds |
| - Multi-site drop offs for bird delivery to final destination |  |
| **Personnel** | |
| - Personnel coming onto farm (crossing PBA) | - LOS Protocol |
| - Personnel going into pens/houses (crossing LOS) | - PBA Protocol |
| - Customers coming onsite (but not crossing PBA)) | - Industry specific tasks required of a flock technician/care taker |
| - 3rd party service personnel going into pens/houses (crossing LOS) | - Division of labor |
| - Veterinary Personnel |  |
| **Equipment** | |
| - Breeder Equipment | - Equipment Sharing |
| - Hatchery equipment | - C&D of equipment |
| - Bird Transport Equipment | - Use of Disposable Equipment |
| - Customer provided Equipment |  |
| **Vehicles** | |
| - Third party vehicle usage for bird delivery | - C&D of vehicles |
| - Deliveries and associated vehicles |  |
