# Supplementary material for: Analysis of geographic location and pathways for influenza A virus infection of commercial upland game bird and conventional poultry farms in the United States of America
Source: BMC Vet Res. 2019 May 14;15:147. doi: 10.1186/s12917-019-1876-y (PMC6518635; doi:10.1186/s12917-019-1876-y)
Supplement: Supplementary file 2 — Table S2. List of production practices compared between industry groups (i.e., commercial upland game bird industry and conventional poultry). The data within this supplementary table outlines which production practices that were compared between industry groups, broken down into the categories of Husbandry, Marketing, Personnel, Equipment, and Vehicles. Within each category, subcategories are defined as appropriate. Categories and subsequent subcategories were derived from information outlined in the Poultry Industry Manual [12]. (DOCX 36 kb) [file 12917_2019_1876_MOESM2_ESM.docx]

Table S2. Complete comparison* of industry group (i.e. commercial upland game bird industry and conventional poultry) practices derived from literature and subject matter experts.

| Husbandry/Management Practices | | | |
| --- | --- | --- | --- |
|  |  |  |  |
| General Practice | Same or Different | Commercial Upland Game Bird Production (Pheasant, Chukar, Bobwhite Quail) | Conventional Confinement Poultry Production (Turkeys, Chickens) |
| Breeder Housing | Different | **Pheasants:** Pens // **Chukar:** outdoor wire floor cages // **Quail:** Confined houses (Ernst 2007, Wallner-Pendelton & Hulet, 2013, Dozier et al. 2010) | Confined houses (Barnes, et al. 2013, Trampel et al. 2013, Trampel, 2013) |
| Breeder Location | Different | Breeder housing onsite with hatcheries, brooder buildings, and growing pens. (Personal Communication, Secure Upland Gamebird Supply Plan Working Group, 6/7/18) | Vertical integration. Breeders have their own separate premises, almost always with no hatcheries, types of birds, or processing plants onsite. (Trampel et al. 2013, Barnes et al. 2013) |
| Hatching Eggs | |  | |
| Collection of Hatching Eggs | Different | Collected by hand typically 3 to 4 times a day depending on the size of pens (can be upwards of 6 times a day for very large operations (i.e. MacFarlane Pheasants). Employees get around in pens using golf carts. 95% of eggs are typically found in shaving-filled hatch boxes but others are found on the ground. (Personal Communication, Secure Upland Gamebird Supply Plan Working Group, 6/7/18, Wallner-Pendelton & Hulet, 2013, Dozier et al. 2010) | Eggs collected by hand or roll-down system. (Barnes, et al. 2013, Trampel et al. 2013) |
| Care/cleaning of Hatching Eggs | Different | **Pheasant eggs:** Manual process. Eggs washed in crates and set into washers (no automated washing systems unless it's a very large operation). Eggs then rinsed via spraying down by hand. **Chukar eggs:** Not washed at all since they are collected from wire cages. (Personal Communication, Secure Upland Gamebird Supply Plan Working Group, 6/7/18) | Eggs washed and sanitized using automatic system. held in a storage room with humidity, temperature, CO2, and oxygen levels controlled appropriately to control egg sweating and maintain highest hatching success. Only clean nest eggs used for hatching. Minor variations dependent on scale of operation and resources. (Trampel et al. 2013) |
| Incubation process | Same | Same process (Eggs placed in automated incubators that maintain proper temperature, humidity, ventilation, egg turning, and egg orientation) with minor variations (hand turning eggs, etc.) dependent on species, scale of operation, and resources available. (Dozier et al. 2010, Pennsylvania State University, 2004a&b, Geaumont, et al. 2016, Ernst, 2007, Trampel et al. 2013, Barnes et al. 2013) | |
| Examination of Eggs | Different | Visually examined by hand. Rarely candled, (Personal Communication, Secure Upland Gamebird Supply Plan Working Group, 6/7/18), but strong lights are needed if candling is performed due egg shell color (Geaumont, et al. 2016, Dozier et al. 2010). | Automated candling, *in ovo vaccination*, transfer to hatchers (Trampel et al. 2013) |
| Transfer of Eggs to Hatchery | Different | Eggs moved breeder pens to cleaning to storage to hatchers without leaving the premises. (Breeders and hatcheries onsite together). (Personal Communication, Secure Upland Gamebird Supply Plan Working Group 6/7/18, Dozier et al. 2010) | Eggs collected, sanitized, and stored/packed then moved to off-site hatchery via truck. (Trampel et al. 2013) |
| Hatchery set up | Same | Same process (Cleaned regularly via fumigation/fogging. Appropriate flow patterns and ventilation systems in place to reduce backflow contamination of clean areas, aka Lines of separation between areas maintained with minor variations dependent on scale of operation and resources. (Ernst, 2007, Trampel et al. 2013, Personal Communication, Secure Upland Gamebird Supply Plan Working Group 6/7/18) | |
| Brooding Components | |  |  |
| Sources of breeders | Different | 80% of flocks are closed. Eggs are sourced from a producer's own breeder flocks. (Personal Communication, Secure Upland Gamebird Supply Plan Working Group, 2016) | Different levels of breeders in broilers/turkey/layer: Elite, great grandparent, grand parent, and parent farms. Eggs from breeder farm to hatchery, day old chicks go from hatchery to either next level of breeder farm or to grow out farm (birds brooded in same barn they are grown to finish/adult weight in). (Trampel, et al 2013a, Barnes, et al. 2013). |
| Brooder building location | Different | Brooder buildings are typically onsite with hatcheries, breeder pens, and growing pens. (Personal Communication, Secure Upland Gamebird Supply Plan Working Group 6/7/18) | Broilers: Brooding occurs in grow out building. Turkeys and Layers: brooding occurs on separate premises at poult or pullet farms, depending on the industry (Trampel, 2013 & Barnes et al 2013). |
| Age groups in brooder premises | Different | Two different age groups will exist in UG brooding facilities, if a Room A/Room B facility is in place: 2 age groups. If single room facility, single age group in and out. (Personal Communication, Secure Upland Gamebird Supply Plan Working Group, 6/7/18) | Single age group in broiler barns and farms (Trampel et al 2013a) |
| Brooding set up (heat sources, light/ventilation, brooder prep) | Same | Same process (either floor brooding requiring a consistent heat source (typically radiant [infrared heaters], litter, set up adjustment as birds, etc. OR battery cage brooding used in some UG and laying pullets) with minor variations dependent on species, scale of operation, and resources. (Dozier et al. 2010, Pennsylvania State University, 2004a&b, Geaumont, et al. 2016, Ernst, 2007, Wallner-Pendelton & Hulet, 2013, Trampel et al. 2013, Trampel, 2013, Barnes et al. 2013) | |
| Use of litter in brooding | Same | Similar usage for brooding birds (chicks raised on litter) with minor variation based on species, scale of operation, and resources. (In broilers, the practice of using “built up” litter is mentioned, not stated in other industries, occasionally used in UG, but not typically). (Geaumont, et al. 2016, Ernst, 2007, Wallner-Pendelton & Hulet, 2013, Trampel et al. 2013, Trampel, 2013, Barnes et al. 2013) | |
| Grower Housing Components | |  |  |
| Growing Housing set up | Different | **Pheasants:** Pens // **Chukar:** outdoor wire floor cages // **Quail:** Pens (Dozier et al. 2010, Geaumont, et al. 2016, Pennsylvania State University, 2004a&b, Ernst, 2007, Wallner-Pendelton & Hulet, 2013) | Confined houses (variations of indoor cages i.e. battery, aviary, etc. in layers) (Barnes, et al. 2013, Trampel et al. 2013, Trampel, 2013) |
| Use of Cover Crops | Different | Cover crops used (with purpose of reducing aggression and general stress among birds) (Pennsylvania State University, 2004a&b, Geaumont, et al. 2016, Ernst, 2007, Wallner-Pendelton & Hulet, 2013) | Not used in indoor buildings (not applicable) (Barnes, et al. 2013, Trampel et al. 2013, Trampel, 2013) |
| Use of litter in growing birds | Different | Not used in outdoor pens/cages (Pennsylvania State University, 2004a&b, Geaumont, et al. 2016, Ernst, 2007, Wallner-Pendelton & Hulet, 2013) | Used in breeder birds, broilers, poults, and market turkeys (Barnes, et al. 2013, Trampel et al. 2013) |
| Water Fountains and Feeders | Different | Floats pans, bowl fountains, and plastic bowl fountains located outside in pens/outdoor cages (Geaumont, et al. 2016, Ernst, 2007, Wallner-Pendelton & Hulet, 2013) (some operations use automatically refilling water system, not applicable in the winter though, because of cold temperatures) (Personal Communication, Secure Upland Gamebird Supply Plan Working Group, 6/7/18) | Closed water system drinkers (nipple drinkers) located inside houses (Barnes, et al. 2013, Trampel et al. 2013, Trampel, 2013) |
| Artificial lighting usage | Different | Lighting used only in breeders for egg laying stimulation. Outdoor lamp posts over pens. (Ernst, 2007) | Lighting used in growing birds for optimal growth/production and breeders for egg laying stimulation and for turkey toms to stimulate sexual activity. (Barnes, et al. 2013, Trampel et al. 2013) |
| Density | Different | Chukar 3 to 5 sq ft/bird ; Pheasant 10 to 12 sq ft/bird ; Quail 3 to 4 sq ft/bird (Pennsylvania State University, 2004a&b; Ernst, et al. 2007). | Broilers 0.5 to 1 sq ft/bird (3-8 lbs) (Trampel et al. 2013) Turkey hens 2.5 sq ft/bird (14-18 lbs) ; Turkey toms 4 sq ft/bird (40-44 lbs) (Barnes et al. 2013) |
| Air Quality Control/Ventilation Control | Different | Open/outdoor pens, no artificial no ventilation control. (No fans or curtains used). (Personal Communication, Secure Upland Gamebird Supply Plan Working Group, 6/7/18) | Houses use 1) natural ventilation (open-sided housing) controlled via curtain adjustments and circulation fans. or 2) power (tune) ventilation controlled via electric exhaust fans and computerized environmental control systems. (Trampel et al. 2013) |
| Temperature Control | Different | Open/outdoor pens, no artificial temperature control (no heat lamps used, propane heating components) (Personal Communication, Secure Upland Gamebird Supply Plan Working Group, 6/7/18) | Broilers: Convection heating systems (forced air heaters) used after 14 day brooding. Computerized environmental control system controls the temperature. (Trampel et al. 2013) |
| Feed | |  | |
| Type of feed | Same | Similar nutritionally optimized pelleted, mashed, crumbled diet with species-specific variations for nutritional requirements. Grit used depending on the species ability to digest (used in UG and sometimes turkeys) (Ernst, 2007, Trampel et al. 2013, Trampel, 2013, Barnes et al. 2013) | |
| Feed source | Same | Both industry groups commonly utilize either their own feed mills or company feed mills (Personal Communication, Secure Upland Gamebird Supply Plan Working Group, 5/31/18) | |
| Feed refilling method | Different | Manual refilling of food in pens. (Geaumont, et al. 2016, Ernst, et al. 2007). | Computerized in layers, automated feed refill systems in place. (Trampel, et al. 2013, Trampel, 2013) |
| Other | |  |  |
| Movement of birds from brooder set up to grower set up | Different | Birds moved out of buildings and into pens at roughly 6 weeks of age (Personal Communication, Secure Upland Gamebird Supply Plan Working Group, 9/23/16) | Broiler brooded and grown in same building, no movement (Trampel et al. 2013) Turkey poults and layer pullets moved to different premises (Trampel, 2013 and Barnes et al, 2013). |
| Bird Catching Method | Different | No contract/outside crews used, all local labor. (Personal Communication, Secure Upland Gamebird Supply Plan Working Group 5/31/18). Birds are most often herded into small areas and are caught by hand or using a landing net. Sometimes funneled into alleyways and into a shoot where they are picked up one by one. Either loaded directly into crates, are either loaded directly into the trailer or transported to trailer first. (Personal Communication, Secure Upland Gamebird Supply Plan Working Group, 6/7/18) | Use of bird catching crews (7 to 10 people) (contracted or company employed with catching being their specific job) birds are caught by hand. Or use of automated catching machines. Turkey loaders used in turkey industry (“cooper” crews used). (Barnes, et al. 2013, Trampel et al. 2013) |
| Bird Depopulation Methods | Same | Similar process (foam, CO2, cervical dislocation) dependent on housing set up and need to herd birds into manageable areas for depopulation (USDA 2015, Wallner-Pendelton & Hulet, 2013) | |
| Dead Bird Disposal | Same | Same process (composting, rendering, burial, and incineration) with variations dependent on scale/set up of operation and resources (Trampel et al. 2013, Barnes, et al. 2013, Ernst, 2007, Geaumont, et al. 2016, Pennsylvania State University, 2004a&b) | |
| Pest Control | |  |  |
| Rodent Control | Same | Same process (Bait stations and traps) with minor variations dependent on scale of operation and resources (Trampel et al. 2013, Barnes, et al. 2013, Ernst, 2007) | |
| Wild bird Control | Different | Netting, fencing (Dozier et al. 2010, Pennsylvania State University, 2004a&b, Geaumont, et al. 2016, Ernst, 2007, Wallner-Pendelton & Hulet, 2013) | Sealed houses that limit wild bird access (Trampel et al. 2013, Barnes, et al. 2013) |
| Predator Control | Different | Preventative measures: Netting, fencing. Active measures: Traps or elimination. Predator losses can occur. (Dozier et al. 2010, Pennsylvania State University, 2004a&b, Geaumont, et al. 2016, Ernst, 2007, Wallner-Pendelton & Hulet, 2013) | Sealed houses that eliminate predator access (Trampel et al. 2013, Barnes, et al. 2013) |
| Integration of Production Stages | Different | Industry occurs on the farm level. Breeder, hatchery, brooding, growing facilities often on one premises. Very few are vertically integrated . Closed flocks, 80% of commercial farms (Wallner-Pendelton & Hulet, 2013, Personal Communication Secure Upland Gamebird Supply Plan Working Group, 2016) | Integration occurs on the industry level and is vertically integrated (Trampel et al. 2013, Barnes, et al. 2013) |
| C&D Process between batches of birds | Different | No C&D happens at in during short downtime if pen segments are used twice in a season. C&D that occurs between seasons includes mowing (if possible) and C&Ding of waterers/water systems and feed containers. (Personal Communication, Secure Upland Gamebird Supply Plan Working Group, 6/7/18) | Litter decaked and remaining litter made friable. Flushing and disinfection of water lines and feed lines cleaned. External maintenance of entryways and outside of buildings (vegetation around outside of building sprayed, bait traps replenished). (Barnes et al. 2013) |
| Complete C&D process used for post depopulation | Different | Destroy all disposable and difficult to clean items. Timing and dry cleaning + wet cleaning of all surfaces and parts of house. Complete disinfect using various methods (Heating, wet disinfection, or fumigation). (USDA, 2016). Differences based on housing (netting destroyed [or sun dried and left out to keep out wild birds from potentially spreading virus → preformed in the ID 2008 UG outbreak], cover crops mowed, etc.) (Frame & Simmunich, 2011) | Destroy all disposable and difficult to clean items. Timing and dry cleaning + wet cleaning of all surfaces and parts of house. Complete disinfect using various methods (Heating, wet disinfection, or fumigation). (USDA, 2016). All surfaces in a barn are completely disinfected. |
| Seasonal production | Different | Breeding, hatching, brooding, and growing all have different seasonal production periods and downtime. Mature birds only grown from August- April. (Wallner-Pendelton & Hulet, 2013, Personal Communication Secure Upland Gamebird Supply Plan Working Group, 2016) | Seasonality not a factor in broilers and turkeys because of maximum outputs achieved via vertical integration and the standard use of confinement systems (Ollinger, et al. 2000) |
|  | | | |
|  |  |  |  |
| Marketing | | | |
|  |  |  |  |
| Turnover rate of market bird flocks | Different | In pens for 16 to 22 weeks. Once or twice per year only 25% of penlets (i.e. segment of a pen) used twice. (Personal Communication, Secure Upland Gamebird Supply Plan Working Group, 5/31/18) | Depends on species:  5.5 times/year for Broilers 2.8 times/year for Turkeys  (USDA, 2007) |
| Turnover rate in brooders |  | Use either all-in-all-out system or Room A/RoomB system for brooder buildings. | 5.5 times/year for Broilers since birds are brooded in the same buildings that they finish in (USDA, 2007). Turkeys: Depends on the system, for single age "brood grow out farm" 2-3 times/year. If three-stage set up: about 8 times/year assuming no down time. (Barnes, et al. 2013) |
| Number of premises involved in production system chain (egg to adult bird) | Different | All components typically occurring on one premises (0 moves) (Personal Communication, Secure Upland Gamebird Supply Plan Working Groups, 2016, 5/31/18, 6/7/18) | **Broilers**: Breeder farm --(egg moving)--> Hatchery --day old chick moving-->Growout farm **(2 moves)** // **Turkeys:** Breeder farm --(egg moving)--> Hatchery --day old chick moving-->poult farm --(poult moving)--> growout farm **(3 moves)**  // **Layer:** Breeder farm Breeder farm --(egg moving)--> Hatchery --day old chick moving--> Pullet farm --(pullets moving)-->Layer facilities **(3 moves)** (Trampel et al. 2013, Trampel, 2013, Barnes et al 2013) |
| Adding additional birds to pens or houses | Different | No instance where additional birds would be added to a pen (Personal Communication, Secure Upland Gamebird Supply Plan Working Group 5/31/18) | Spiking apparent in broiler industry (Trampel et al. 2013) |
| Partial farm removals | Same | Similar processes. Multi-age premises (turkeys and layers) participate in this practice ensuring that there are always birds producing on the premises (USDA, 2000, USDA, 2011) Common practice in the UG industry (Personal communication, Secure Upland Gamebird Supply Plan Working Group, 2016.) | |
| Partial flock removals | Different | Sell small batches of birds at a time. Pens aren't cleared out all at once. (Personal communication, Secure Upland Gamebird Supply Plan Working Group, 2016, Observed in unpublished mortality data) | Vast majority of commercial poultry farms operate as all-in, all out operations (Pepin, 2014) This includes all broiler operations and some turkey operations. (USDA, 2011). In commercial multi-age poultry operations, flocks are loaded out with downtime between flocks in a barn, indicating all birds in a flock are removed at once.  (USDA, 2000, Barnes, et al. 2013) |
| Flock/house/pen down time | Different | If pen segments are used once per season: There is an annual downtime of roughly 34 weeks for most pens. If pen segments are used more than once per season: Downtime is only about 1 week between uses DURING the season (Personal Communication, Secure Upland Gamebird Supply Plan Working Group 5/31/18) Every upland gamebirds farm requires two months of downtime for market bird pens in order to regrow cover crops. For specific farms 75% of farms have six months of downtime and some pens have up to eight months of downtime. (Personal Communication, Secure Upland Gamebird Supply Plan Working Group 7/5/18) | Minimum downtime between flocks in some industries is roughly two weeks (turkeys, egg layers, broilers) (Barnes, et al. 2013, USDA, 2000, Hubbard, 2016) |
| Distances birds are transported to market | Different | Sales groups of birds can travel on average anywhere from 100 to 1000 miles depending on the location of the farm. (Personal Communication, Secure Upland Gamebird Supply Plan Working Group 5/31/18) | Most broilers (91.2%) and turkeys (88.2%) shipped within state lines to processing according to one study (USDA, 2011) |
| Multi-site drop offs for bird delivery to final destination | Different | Occurs regularly because of scale of batches being small. Operations want to be efficient. (Wallner-Pendelton & Hulet, 2013) | All birds go to one processing plant (Personal Communication, Cardona) |
| Onsite purchases of birds | Different | Occurs on almost all farms (Personal communication, Secure Upland Gamebird Supply Plan Working Group, 2016) | Highly unlikely to happen because of closed marketing systems. Not involved in livestock markets. (Trampel et al. 2013, USDA 2011). |
| Market channel for live-birds (End destination for sold birds) | Different | Primarily Live-bird release | Processing |
| Number of customers/contracts | Different | Sell to multiple customers. Not dependent on one contract/customer to buy birds. (Personal Communication, Secure Upland Gamebird Supply Plan Working Group 5/31/18) | Typically in contract with one processor. (Personal Communication, Carol Cardona, 5/31/18) |
| Seasonal customer base | Different | Hunting preserves and individual hunters/dog trial participants purchase birds only during the designated hunting seasons. (Personal Communication, Secure Upland Gamebird Supply Plan Working Group 5/31/18) | Year-round production system aiming for maximum output regardless of season. Customers expecting product year-round. (Personal Communication, Carol Cardona, 5/31/18) |
| Number of other vendors dropping off at bird delivery site | Different | Hunting preserves/primary customer buying birds source from a single or a few vendors, and thus only a single or few vendors will drop off at a given site. (Personal Communication, Secure Upland Gamebird Supply Plan Working Group, 5/31/18) | Processors will receive batches of birds from the numerous company and independent farms that they contract with. (Personal Communication, Carol Cardona, 5/31/18) |
|  | | | |
|  |  |  |  |
| Personnel | | | |
|  |  |  |  |
| Personnel coming onto farm (crossing PBA) | Different | Farm Managers and Flock Caretakers serving multiple roles (Caring for birds, performing maintenance, delivering birds, c&d etc.) Consulting veterinarians. (Personal Communication, Secure Upland Gamebird Supply Plan Working Group 5/31/18, 6/7/18) | Additional personnel aside from farm managers and flock caretakers (Land maintenance, cleaning crews, catching crews, vaccination crews, insemination crews for turkeys, company veterinarians, electronic system maintenance personnel, etc). (Barnes, et al. 2013, Trampel et al. 2013, Trampel, 2013) |
| Personnel going into pens/houses (crossing LOS) | Different | Farm Managers and Flock Caretakers serving multiple roles (Caring for birds, performing maintenance, delivering birds, c&d etc.) Consulting veterinarians**.** (Personal Communication, Secure Upland Gamebird Supply Plan Working Group, 5/31/18) | Additional personnel aside from farm managers and flock caretakers (Land maintenance, cleaning crews, catching crews, vaccination crews, insemination crews, company veterinarians, etc). (Barnes, et al. 2013, Trampel et al. 2013, Trampel, 2013) |
| Customers coming onsite (but not crossing PBA)) | Different | Customers come onsite for sale (Personal Communication, Secure Upland Gamebird Supply Plan Working Group, 2016) But customers do not cross the PBA (Personal Communication, Secure Upland Gamebird Supply Plan Working Group, 2018) | Customers do not come onsite (Personal Communication, Carol Cardona, 5/31/18) |
| 3rd party service personnel going into pens/houses (crossing LOS) | Different | No electrical maintenance needed for inside the actual pens (Personal Communication, Secure Upland Gamebird Supply Plan Working Group, 5/31/18) | Houses have automated systems and computer controllers to control ventilation, lighting, and temperature. Such systems require specialized maintenance and maintenance crews. (Trampel et al. 2013) |
| LOS Protocol | Different | LOS protocols in place (Pennsylvania State University, 2004a&b), but much more limited. Outdoor systems have inherent limitations to LOS protocols because of their openness. Barn-specific PPE is not used when entering pens. (Personal Communication, Secure Upland Gamebird Supply Plan Working Group 5/31/18) | Danish entryways, Barn-specific clothing/footwear, PPE, footbaths, hand sanitizer all utilized in protocol. (Trampel et al. 2013, Trampel, 2013, Barnes et al. 2013). |
| PBA Protocol | Same | Similar recommendations from industry literature based on disease transmission (avoiding contact with wild birds and other poultry 24hrs/48hrs/etc before coming onto premises, etc.) (Ernst, 2007, Wallner-Pendelton & Hulet, 2013, Barnes, et al. 2013, Trampel et al. 2013, Trampel, 2013) | |
| Veterinary Personnel | Different | Use of third party veterinarians. Often times veterinarians that attend to one UG farm, usually do not attend to other UG farms or other poultry farms (reason associated with geographic isolation of UG farms from other farms). (Personal Communication, Secure Upland Gamebird Supply Plan Working Group, 6/7/18) | Company veterinarian typically tends to flocks. Is in charge of all of the flocks for a single company. Field technicians carry out veterinary tasks under the supervision of the company veterinarian. (Trampel et al. 2013) |
| Industry specific tasks required of a flock technician/care taker | Different | Installing specs (aka peeping birds). Outdoor pen maintenance. Manually refilling feed and water stations (especially in the winter when automated systems can't function in extreme temperatures). (Personal Communication, Secure Upland Gamebird Supply Plan Working Group, 6/7/18) | All: Break trimming, sexing, etc. Turkeys: Poult tending, insemination Layers: Vaccination. (Trampel et al. 2013, Trampel, 2013, Barnes et al. 2013) |
| Division of labor | Different | Employees typically do a myriad of tasks depending on the season. Only specific employee type would be a designated hatchery worker, but even then, in non-growing season, these personnel are often times performing other job functions. (Personal Communication, Secure Upland Gamebird Supply Plan Working Group, 6/7/18) | Employee types have specific tasks. (Beak trimming crew, vaccination crew, flock technicians, flock care taker, ground maintenance, catching crew, hatchery worker (In part due to high level of vertical integration and separation of operation on different premises). (Trampel et al. 2013, Trampel, 2013, Barnes et al. 2013) |
|  | | | |
|  |  |  |  |
| Equipment | | | |
|  |  |  |  |
| Breeder Equipment | Different | Outdoor nest boxes (Ernst, 2007, Wallner-Pendelton & Hulet, 2013) | Nest boxes, insemination/semen collection equipment and crews needed for turkeys (Barnes, et al. 2013, Bakst & Dymond, 2013) |
| Hatchery equipment | Same | Similar equipment (setters and hatchers) with minor variations dependent on scale of operation and resources (Ernst, 2007, Wallner-Pendelton & Hulet, 2013, Barnes, et al. 2013, Trampel et al. 2013, Trampel, 2013) | |
| Bird Transport Equipment | Different | Various crates that are manually loaded into truck beds or trailers (Ernst, 2007, Wallner-Pendelton & Hulet, 2013) | Use large packing units moved onto trucks via forklift (Broilers) Use large coop units that turkeys are loaded into via turkey loading machine. (Barnes, et al. 2013, Trampel et al. 2013) |
| Customer provided Equipment | Different | Customers will bring own crates to get birds onsite or purchase disposable crates (Personal Communication, Secure Upland Gamebird Supply Plan Working Group, 2016) | Customers wouldn’t come onto farms directly because of the closed market system. (Trampel et al. 2013) |
| Equipment Sharing | Different | No equipment sharing between any farms for the UG industry. UG farms typically have all their own equipment. (Personal Communication, Secure Upland Gamebird Supply Plan Working Group, 5/31/18) | About one-fourth of farms with off-farm processing used racks/pallets that were also used by other companies (USDA, 2011). |
| C&D of equipment | Different | Because there is limited sharing of equipment, C&D is not as formalized. Pressure washers used to spray equipment down. (Personal Communication, Secure Upland Gamebird Supply Plan Working Group, 5/31/18) | Similar processes recommended across conventional industries (Ernst, 2007, Wallner-Pendelton & Hulet, 2013, Barnes, et al. 2013, Trampel et al. 2013, Trampel, 2013) with some variation of exact C&D process depending on the individual farm. |
| Use of Disposable Equipment | Same | Similar. Disposable crates and egg flats used sometimes (Personal Communication, Secure Upland Gamebird Supply Plan Working Group, 5/31/18) Trampel, 2013) Variation dependent on resources available to operations. | |
|  | | | |
|  |  |  |  |
| Vehicle | | | |
|  |  |  |  |
| Third party vehicle usage for bird delivery | Different | Very rarely occurs. 99% of farms don’t use third party (shared) vehicles for deliveries (Personal Communication, Secure Upland Gamebird Supply Plan Working Group, 2016) | Broiler and turkey farms most commonly used vehicles dedicated to the company only to transport birds to slaughter. Over 80 percent of table-egg companies used vehicles that were also used by other companies (USDA, 2011) |
| Deliveries and associated vehicles | Same | Similar types of deliveries occurring: feed, litter, mail, propane, etc. (Personal Communication, Secure Upland Gamebird Supply Plan Working Group, 2016, Wallner-Pendelton & Hulet, 2013, Barnes, et al. 2013, Trampel et al. 2013) | |
| C&D of vehicles | Same | Similar process (spray off and disinfect wheels and undercarriage of vehicles before coming on and off farms) (Ernst, 2007, Barnes, et al. 2013, Trampel et al. 2013, Trampel, 2013) | |

*Note that while frequencies and detailed observations were recorded, the qualitative analysis solely focused upon absence vs presence of different practices rather than the frequencies related to specific practices.

**References**

Bakst, M. R., and J. S. Dymond. Artificial Insemination in Poultry. In: Success in Artificial Insemination. A. Lemma, ed. IntechOpen. pp 175-195. 2013.

Barnes, H. J., S. E. Tilley, and M. P. Martin. Turkey Industry. In: Poultry Industry Manual. pp 46-97. 2013.

Dozier, W. A., Brmawell, K., Hatkin, J., & Dunkley, C. (2010). Bobwhite Quail Production and Management Guide. (pp. 1-8): The University of Georgia Cooperative Extension

Ernst, R. A. Raising Game Birds. UCANR Publications. 2007.

Frame, D. D., and M. M. Simunich. Biosecurity Challenges on a Multi-Species Game Bird Farm with Detectable Avian Influenza Subtype H5N8 Exposure. In: 60th Western Poultry Disease Conference. Sacramento, California. pp 63-65. 2011.

Geaumont, B., Sedivec, K., & Field, A. Raising Pheasants. In (pp. 1-12): North Dakota State University.

Hubbard, L. Management guide: Broiler.

Ollinger, M., J. MacDonald, and M. Madison. Structural Change in U.S. Chicken and Turkey Slaughter. In. Economic Research Service, U.S. Department of Agriculture, <http://ers.usda.gov/publications.> pp 1-48. 2000.

Pepin, K., E. Spackman, J. Brown, K. Pabilonia, L. Garber, J. Weaver, D. Kennedy, K. Patyk, K. Huyvaert, and R. Miller. Using quantitative disease dynamics as a tool for guiding response to avian influenza in poultry in the United States of America. Preventive veterinary medicine 113:376-397. 2014.

Pennsylvania State University. (2004a). Agriculture Alternatives: Partridge Production. (pp. 1-6): College of Agricultural Sciences Agricultural Research and Cooperative Extension.

Pennsylvania State University. (2004b). Agriculture Alternatives: Pheasant Production. (pp. 1-6): College of Agricultural Sciences Agricultural Research and Cooperative Extension.

Trampel, D. W. Egg Industry. In: Poultry Industry Manual. United States Department of Agriculture. pp 98-129. 2013.

Trampel, D. W. F., Malea, K. Evans, and H. J. Barnes. Broiler Industry. In: Poultry Industry Manual. United States Department of Agriculture. pp 4-45. 2013.

USDA. Part II: Reference of  1999 Table Egg Layer Management  in the U.S.  . In., https://www.aphis.usda.gov/animal_health/nahms/poultry/downloads/layers99/Layers99_dr_PartII.pdf. 2000.

USDA. National Agricultural Statistics Service, 2007. Census of Agriculture. In., [http://www.agcensus.usda.gov.](http://www.agcensus.usda.gov./) 2007.

USDA. Structure of the US Poultry Industry, 2010. In., [https://www.aphis.usda.gov/.](https://www.aphis.usda.gov/) 2011.

USDA. FY2016 HPAI Response:  Cleaning & Disinfection Basics  (Virus Elimination). In. pp 1-2. 2016.

Wallner-Pendelton, E., and R. M. Hulet. Gamebird Industry. In: Poultry Industry Manual. United States Department of Agriculture. pp 130-149. 2013.
